# Treatment-Related Mechanisms of Tibetan Medicine *Terminalia chebula* (TC) Aqueous Extract Against Mouse Gastroenteritis Caused by Yak-Origin *Salmonella* Determined Using Intestinal Microbiome Analysis and Metabolomics

**DOI:** 10.3390/ani15050755

**Published:** 2025-03-06

**Authors:** Dengyu Li, Kaiqin Zhang, Xiaofeng Xue, Zhanchun Bai, La Yang, Jingjing Qi, Sizhu Suolang

**Affiliations:** 1 College of Animal Science, Tibet Agricultural and Animal Husbandry University, Nyingchi 860000, China; 202100201101@stu.xza.edu.cn (D.L.);; 2 Shanghai Veterinary Research Institute, Chinese Academy of Agricultural Sciences China, Shanghai 200241, China; 3 Fourteenth Five-Year Plan China Agricultural Rural Ministry Key Laboratory, Jointly Built by the Ministry and Provincial Government, Nyingchi 860000, China

**Keywords:** *Terminalia chebula* (TC), gastroenteritis, gut microbiome, non-targeted metabolism, *Salmonella*

## Abstract

This study aimed to evaluate the therapeutic effect of *Terminalia chebula* (TC) on Tibetan yak-origin *Salmonella*-induced diarrhea and dysentery in mice. The levels of pro-inflammatory cytokines (IL-1β, IL-6, IL-8, and TNF-α), anti-inflammatory cytokines (IL-4 and IL-10), and the oxidative stress markers malondialdehyde (MDA), superoxide dismutase (T-SOD), total antioxidant capacity (T-AOC), reduced glutathione (GSH-PX), and catalase (CAT) in the serum of mice were measured using ELISA kits. Using microbial diversity sequencing and non-targeted metabolomics detection techniques, the relevant mechanisms of TC treatment in a mouse *Salmonella* infection model were evaluated. The results showed the following: TC can effectively reduce the diarrhea rate; alleviate weight loss caused by *Salmonella* invasion; reduce the pro-inflammatory cytokines IL-1β, IL-6, IL-8, and TNF-α in serum; and increase the concentrations of the anti-inflammatory cytokines IL-4 and IL-10. TC can improve the body’s antioxidant levels to heal the damage caused by oxidative stress and lipid peroxidation. The histological section results show that TC can significantly improve gastric and intestinal tissue lesions and has no toxic effects on the liver and kidneys. 16S rRNA and ITS sequencing analysis suggests that *Lactobacillus*, *Enterorhabdus*, *Alistipes* (bacterial community), *Lodderomyces*, *Saccharomyces*, and *Penicillium* (fungal community) may be key functional microbial communities in TC. Non-targeted metabolomics also suggests that the antibacterial treatment of dysentery with chebulic acid may be related to regulation of the Ras signaling pathway, long-term potentiation, the MAPK signaling pathway, metabolic pathways, and gut microbiome composition. Conclusion: TC has clear clinical efficacy in treating bacterial diarrhea, presenting anti-inflammatory and antioxidant effects. Its roles in regulating the gut microbiome and metabolic pathways and products were determined as the main reason for its therapeutic effect in a mouse gastroenteritis model caused by *Salmonella* infection.

## Simple Summary

*Salmonella* is the main pathogen causing diseases in Tibetan yak on the Qinghai–Tibetan Plateau. Infected animals show symptoms such as sepsis, enteritis, gastroenteritis, and arthritis, leading to slow growth and reduced production performance, which causes significant economic losses for local farmers and herders. This study aims to investigate the treatment mechanism of the Tibetan medicine *Terminalia chebula* (TC) against intestinal enteritis caused by *Salmonella* enterica serovar enteritidis in mice using gut microbiome and non-targeted metabolomics methods. In mice, TC can reduce the diarrhea rate; regulate pro-inflammatory and anti-inflammatory cytokine levels; adjust antioxidant indicators; improve gastric and intestinal lesions; regulate key microbial populations, such as *Lactobacillus* and *Lodderomyces*; and treat *Salmonella*-induced gastroenteritis by changing the metabolite 1,2-Dihydroxy-3-keto-5-methylthiopentene via major pathways, such as the Ras signaling pathway.

## 1. Introduction

The drug *Terminalia chebula* (TC) is made from the ripe fruit of the chebula (*Terminalia chebular Retz*), a plant of the family Eleutherococcus. The fruit of the plant exerts a pharmacological effect. Its name in Mongolian medicine is “Arura”, and it is called “Ma Cai Guo” in Thai. It is native to India and Myanmar and is distributed in Yunnan, Guangdong, Guangxi, and Tibet in China. TC is widely used in traditional Chinese medicine to treat various diseases and is known as the “King of Medicines” in Tibetan medicine [1]. Ayurveda (Indian traditional medicine) has a 5000-year history in India. In this practice, horehound is used to treat various ailments due to its high medicinal value, and it is one of the top-ranked natural medicines among Ayurvedic herbs [2,3]. Modern pharmacological studies have shown that TC extracts have antibacterial, antioxidant, hypoglycemic, antiviral, and anti-inflammatory effects and can kill or inhibit the growth of malignant tumor cells. TC can also reduce the toxicity of aconite [4]. It is used for a variety of purposes: the fruit is used to treat stomach ailments and diarrhea; the dried powder is used as an anti-inflammatory and pain reliever; and TC boiled in water is used to treat mouth ulcers and sore throats [5,6]. The yak is a special species used at high altitudes and is the main breeding livestock and economic source of local farmers and herdsmen. However, yak diarrhea has caused great economic losses for these herdsmen. The vast majority of yak diarrheal diseases in the plateau area are caused by *Salmonella* [7], an enteropathogenic Gram-negative bacillus. *Salmonella* is a very significant pathogen in animal husbandry and public health, posing a major threat to global public health [8].

Young yaks are especially susceptible to *Salmonella*, and the main lesions caused by infections are septicemia, gastroenteritis, and local tissue inflammation [9]. *Salmonella* can destroy the intestinal mucosal layer [10], changing the structure of the intestinal cytoskeleton and decreasing the function of the intestinal epithelial cells in absorbing and utilizing nutrients and water, which can lead to gastroenteritis in yaks [11]. *Salmonella* infection is associated with increased levels of pro-inflammatory cytokines, such as IL-1β, IL-6, and IL-8, within living organisms [12]. Salmonellosis leads to significant annual economic losses in yak farming [13]. Although antibiotics are currently used to treat *Salmonella* infections, their widespread use can cause *Salmonella* to develop resistance [14] or even multi-drug resistance. They can also leave toxic residues that threaten human health and the environment [15]. Thus, finding a natural botanical alternative to traditional antibiotics for treating infections caused by *Salmonella* can greatly mitigate the development of *Salmonella* resistance.

Microbial diversity sequencing uses bacterial 16S rRNA genes and fungal ITS genes to assess changes in the abundance, diversity, and dominant populations of gut microbiota [16,17]. Metabolomics applies qualitative and quantitative analyses to identify small molecule metabolites in biological samples [18], and constituents or metabolites have the potential to be used as disease diagnostic biomarkers or alternative targets for disease treatment [19].

Multi-omics is currently being applied in veterinary medicine to analyze correlations between changes in gut microbial species and altered metabolite dynamics in organisms and to elucidate their host protection mechanisms [20]. In this study, microbial diversity sequencing and metabolomics were combined to determine whether and how TC alleviates host gastroenteritis disease by antagonizing its pathogen, *Salmonella*, as well as to investigate other alterations to species in the microbial community composition within the organism. Association analysis can identify key bacterial and metabolic pathways associated with enteritis and determine the mode of action by which TC puts enteritis lesions into remission. The present study provides a theoretical and empirical basis for TC in treating yak salmonellosis and screening natural phytopharmaceuticals that can be used as an alternative to antibiotics for this purpose.

## 2. Materials and Methods

### 2.1. Materials and Chemicals

Drug: Mature fruit of TC (Xizang Medicine Co., Ltd., Lhasa, China).

Reagents: IL-1β, IL-6, IL-8, TNF-α, IL-4, and IL-10 enzyme-linked immunosorbent assay (ELISA) kits (Nanjing Jiancheng Biological Engineering Research Institute, Nanjing, China). We used the antioxidant indicators of malondialdehyde (MDA), T-SOD, total superoxide dismutase (T-AOC), glutathione peroxidase (GSH-PX), and catalase (CAT) (Nanjing Jiancheng Biological Engineering Research Institute, Nanjing, China). Tryptic soy broth (TSB) culture medium, PBS (phosphate buffer solution), normal saline (Thermo Fisher Scientific—CN, Shanghai, China), and all the other chemicals and reagents were of analytical grade.

Experimental Animals: Sixty ICR mice (equal numbers of males and females, aged 4–6 weeks; Shanghai Jiesijie Experimental Animal Co., Ltd., Shanghai, China), weighing 18–20 g, were used.

The strain of enteric *Salmonella* originated between September 2019 and September 2022. It was isolated and identified from fresh dung samples from yaks collected in the main yak breeding areas of the Qinghai–Tibet Plateau in China and preserved at the Center for High-Altitude Animal Infectious Diseases of the Xizang Agriculture and Animal Husbandry University.

### 2.2. Preparation of TC

The dried TC material was first crushed into fine powder, followed by screening through a 20-mesh sieve. Then, the powder was mixed with water in a ratio of 1:10 (*v*/*v*) and subjected to rotary evaporation at 100 °C for two extractions, each lasting 30 min. After extraction, the suspension was centrifuged at 12,000 rpm/min at 4 °C for 10 min, and the supernatant was frozen overnight at −80 °C to obtain dried chebulic extract powder for future use.

### 2.3. Revival of Salmonella

Using the method described in Reference [21], we thawed the *Salmonella* bacteria for recovery. We poured the *Salmonella* freezing solution into a shaking flask containing TSB medium and placed it on a shaking incubator at 180 rpm at a temperature of 37 °C for 8 h. At this time, we used a McFarland turbidimeter to measure the bacterial suspension concentration at a wavelength of 600 nm. We set the D600_nm_ value to 0.6–0.8; based on the results of the preliminary experiment, at this absorbance value, the bacterial concentration was approximately 1.5 × 10^8^ CFU/mL, which was consistent with the 0.5 McFarland standard.

### 2.4. Animal Vaccination and Treatment

The animals used in the experiment were 4–6-week-old, 18–20 g, male and female ICR mice.( Shanghai JieSiJie Laboratory Animal Co., Ltd, Shanghai, China) These mice were acclimated for one week prior to the experiment. The mice were placed in a controlled environment with a temperature of 24 ± 1 °C, humidity of 50% ± 10%, and a light–dark cycle of 12/12 h. These animal experiments were conducted in accordance with the principles of animal care and were approved by the Local Ethics Committee for the Care and Use of Animals at the Xizang Agriculture and Animal Husbandry University. In total, 60 mice were randomly divided into 6 groups, with 12 mice in each group, namely the control (negative–uninfected control), model (positive–infected control), positive (the 5-aminosalicylic acid treatment group), TC low, TC medium, and TC high groups. The entire experiment was conducted according to a complete pharmacological cycle, totaling 14 days.

Before initiating formal TC drug treatment, a mouse model of enteritis was established. The main method involved administering 200 μL of a 5 × 10^4^ CFU/mL *Salmonella* bacteria suspension via gavage to the model, positive, TC low, TC medium, and TC high mice every day, with a total gavage dose of 1 × 10^4^ CFU. The dose was determined in a preliminary experiment as the minimum dose required to induce disease; i.e., the mice exhibited diarrhea but did not die, and histological examination using HE staining revealed abnormalities in the stomach and intestines, while the liver and kidneys showed no pathological changes. After continuous gavage for 3 days, we observed diarrhea and lethargy in the mice, with obvious pathological changes in the stomach and intestines upon dissection, proving that the model had been successfully established. After successfully modeling intestinal inflammation, a treatment experiment was conducted. The positive group was orally administered 200 mg/(kg d) of 5-Aminosalicylic acid (5-ASA). According to the dosage method in the “Pharmacological Research Methodology of Traditional Chinese Medicine”, the adult dose of chebulic myrobalan is approximately 1.242 g/(kg d). The kilogram weight conversion coefficient for mice is 9.1; so, the normal oral dose is 9.1 × 1.242 = 11.302 g/(kg d). This dose was set as the oral dose for the middle-dose group; 5.651 g/(kg d) was set for the low-dose group, and 22.604 g/(kg d) was set for the high-dose group. The control group was orally administered an equal amount of physiological saline, and the experiment lasted for 14 days. Blood and intestinal content samples were collected for further experimental determination.

### 2.5. Physiological Parameter Measurement and Sample Collection

The diarrhea rate of the mice was determined on the last day of the experiment, and weight changes in each group were measured throughout. Once the formal experiment was completed, 0.1 mL of 0.3% pentobarbital sodium solution was used to anesthetize the mice, and 1 mL of whole blood from the eye socket was collected. Then, the mice were euthanized via carbon dioxide inhalation. The samples rested at 37 °C for 2 h. The blood serum was then obtained via centrifugation at 3500 rpm for 10 min, and the colonic content and small intestinal tissue were collected.

### 2.6. Histological Examination of the Intestinal Tissue

Collected intestinal tissues from each group were immersed in 4% paraformaldehyde; then, we performed hematoxylin–eosin (HE) staining to observe the morphological and pathological changes in the intestinal tissues of each group.

### 2.7. In Vivo Safety Evaluation

The collected liver and kidney tissues from each group were immersed in 4% paraformaldehyde solution. Then, hematoxylin–eosin (HE) staining was performed to observe the morphological and pathological changes in the liver and kidney tissues of each group of mice.

### 2.8. Determination of Inflammatory Cytokines in Serum

The levels of pro-inflammatory factors (IL-1β, IL-6, IL-8, and TNF-α) and anti-inflammatory factors (IL-4 and IL-10) in the serum were determined using reagents provided by (NanJing, Jiangsu, China) NanJing JianCheng Bioengineering Institute. In total, 100 μL of the diluted serum sample and 100 μL of horseradish peroxidase-conjugated detection antibody were added to the corresponding precoated ELISA wells. After the colorimetric reaction, the absorbance value at 450 nm was measured using an enzyme-linked immunosorbent assay (ELISA) reader. The IL-1β, IL-6, IL-8, TNF-α, IL-4, and IL-10 contents were calculated based on the standard curve.

### 2.9. Determination of Serum Antioxidant Indicators

The serum malondialdehyde (MDA) levels, total antioxidant capacity (T-AOC), and activities of superoxide dismutase (T-SOD), catalase (CAT), and glutathione peroxidase (GSH-Px) were strictly determined according to the kit’s instructions (NanJing JianCheng Bioengineering Institute, NanJing, Jiangsu, China).

### 2.10. Analysis of 16S rDNA and ITS Metagenomic Sequencing of Fecal Samples

After drug intervention, fecal samples were collected from the model, positive, TC low, TC medium, and TC high groups. These samples were sent to (Shanghai, China) Shanghai Meij Bio-Tech Co., Ltd., for sequencing analysis. Each group had 6 parallel replicates submitted for testing.

In summary, the main steps included sample DNA extraction; the design and synthesis of primers and adapters; PCR amplification and purification of products; quantification and normalization of PCR products; the construction of a PE library; and Illumina sequencing.

According to the instructions of the E.Z.N.A.^®^ soil DNA kit (Omega Bio-tek, Norcross, GA, USA), microbial community total genomic DNA was extracted, and the quality of the extracted genomic DNA was detected via 1% agarose gel electrophoresis. The DNA concentration and purity were determined using a NanoDrop2000 (Thermo Scientific, Massachusetts, MA, USA). Using the DNA extracted above as a template, PCR amplification of the V3-V4 variable region and ITS gene of the 16S rRNA gene was performed using upstream primer 338F (5′-ACTCCTACGGGAGGCAGCAG-3′) carrying a barcode sequence and downstream primer 806R (5′-GGACTACHVGGGTWTCTAAT-3′).

The PCR reaction mixture consisted of the following: 5× TransStart FastPfu buffer, 4 μL; 2.5 mM dNTPs, 2 μL; upstream primer (5 uM), 0.8 μL; downstream primer (5 μM), 0.8 μL; TransStart FastPfu DNA polymerase, 0.4 μL; and template DNA, 10 ng. The total volume was 20 μL. The amplification procedure was as follows: pre-denaturation at 95 °C for 3 min, followed by 27 cycles (95 °C denaturation for 30 s, 55 °C annealing for 30 s, and 72 °C extension for 30 s), then 72 °C stable extension for 10 min, and storage at 4 °C (ABI GeneAmp^®^ 9700 model: Thermo Fisher Scientific, Massachusetts, MA, USA). We used 2% agarose gel to recover the PCR products, purified the recovered products using a DNA gel recovery purification kit (Thermo Fisher Scientific, Massachusetts, MA, USA), and quantified the recovered products using Qubit 4.0 (Thermo Fisher Scientific, Massachusetts, MA, USA).

We used the NEXTFLEX Rapid DNA-Seq Kit (Revvity, Waltham, MA, USA) to prepare the library of the purified PCR products: (1) linker attachment was performed; (2) magnetic beads were used to selectively remove linker self-ligated fragments; (3) the library template was enriched using PCR amplification; and (4) the PCR products were recovered using magnetic beads to obtain the final library. We used the Illumina PE300/PE250 platform for sequencing (Shanghai Meij Bio-Pharmaceutical Technology Co., Ltd., Shanghai, China). The raw data were uploaded to the NCBI SRA database.

### 2.11. Metabolomics Data Analysis of Non-Target Compounds

LC-MS/MS analysis was performed on the sample using a Exactive HF-241 X UHPLC-Q system (Thermo Fisher Scientific, Massachusetts, MA, USA).

Chromatographic conditions: A 3 μL sample was separated using an HSS T3 chromatographic column (Waters, Massachusetts, MA, USA) (100 mm × 2.1 mm i.d., 1.8 µm) and then subjected to mass spectrometry detection. Mobile phase A was 95% water + 5% acetonitrile (containing 0.1% formic acid), and mobile phase B was 47.5% acetonitrile + 47.5% isopropanol + 5% water (containing 0.1% formic acid). The flow rate was 0.40 mL/min, and the column temperature was 40 °C.

Mass spectrometry conditions: The mass spectrometry signal of the sample was collected using the positive and negative ion scanning modes, with a mass scanning range of 70–1050 m/z. The sheath gas flow rate was 50 psi, the auxiliary gas flow rate was 13 psi, the auxiliary gas heating temperature was 425 °C, the capillary temperature was 325 °C, the positive mode ion spray voltage was set to 3500 V, the negative mode ion spray voltage was set to −3500 V, and the normalized collision energy was 20–40–60 eV cyclic collision energy. The first-order mass spectrometry resolution was 60,000, the second-order mass spectrometry resolution was 7500, and data were collected using the DDA mode.

After being uploaded, the LC-MS raw data were imported into the metabolomics processing software Progenesis QI(v2.0) (Waters, Massachusetts, MA, USA) for baseline filtering, peak identification, integration, retention time correction, and peak alignment, ultimately resulting in a matrix of the retention time, mass-to-charge ratio, and peak intensity. At the same time, the MS and MSMS mass spectrometry information was matched with the metabolic public databases HMDB (http://www.hmdb.ca/ (accessed on 20 December 2023)) and Metlin (https://metlin.scripps.edu/ (accessed on 20 December 2023)), as well as the Meijer in-house library, to obtain metabolite information.

After searching the database, the data matrix was uploaded to the Majorbio Cloud Platform (cloud.majorbio.com(accessed on 25 December 2023)) for analysis. First, the data matrix was preprocessed as follows: The data matrix was cleaned using the 80% rule to remove missing values, i.e., retaining at least 80% of the non-zero values in each sample group and then filling in missing values (the minimum value in the original matrix is used to fill in missing values). To reduce errors caused by sample preparation and instrument instability, the response intensity of the sample spectrum peaks was normalized using the total sum normalization method, and a normalized data matrix was obtained. At the same time, the variables with relative standard deviations (RSDs) greater than 30% in the QC sample were deleted, the data matrix was log10-transformed, and the final data matrix used for the subsequent analysis was obtained.

Secondly, the preprocessed data matrix was analyzed via principal component analysis (PCA) and orthogonal partial least squares discriminant analysis (OPLS-DA) using the ropls package (Version 1.6.2) in R language, and model stability was evaluated via 7-fold cross-validation. Significant metabolites were selected based on the variable weight values (VIPs) and Student’s *t*-test *p*-values obtained from the OPLS-DA model. Metabolites with VIP > 1 and *p* < 0.05 were considered significant metabolites.

Differentially expressed metabolites were annotated with metabolic pathways using the KEGG database (https://www.kegg.jp/kegg/pathway.html (accessed on 12 January 2024)) to obtain the metabolic pathways in which differentially expressed metabolites were involved. The pathway enrichment analysis was performed using the Python software package scipy.stats (v1.4.1), and the most relevant biological pathways were obtained using Fisher’s exact test.

### 2.12. Data Statistics

The quantitative data were expressed as mean ± standard deviation. The difference analysis compared to the model group was conducted using SPSS Statistics 22.0 in accordance with the Student’s *t*-tests. *p*-values < 0.05 indicated the significance level.

## 3. Results

### 3.1. Mouse Diarrhea Rate and Weight Changes

**Figure 1:**
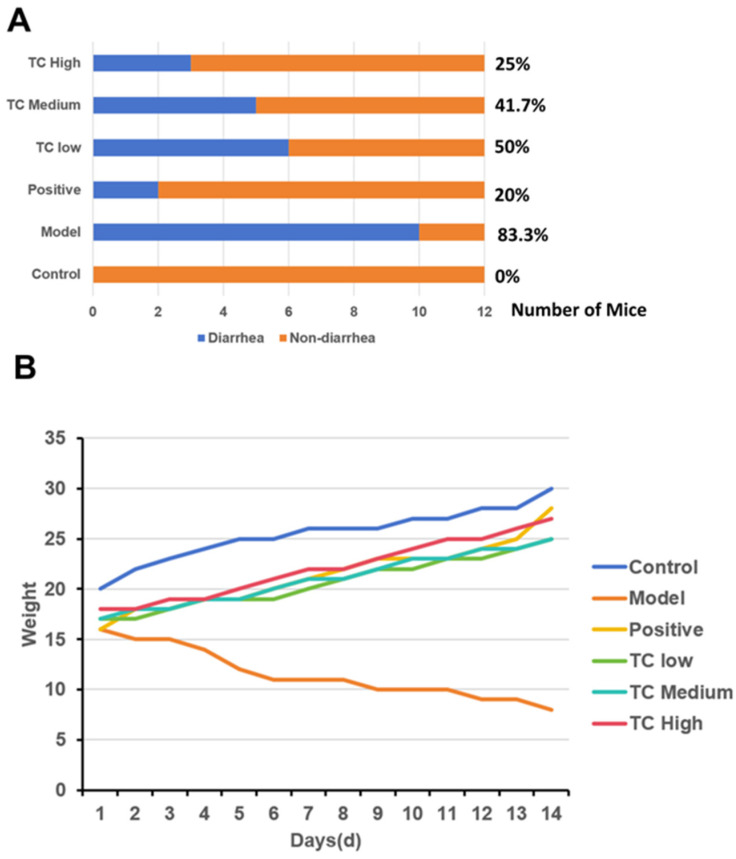

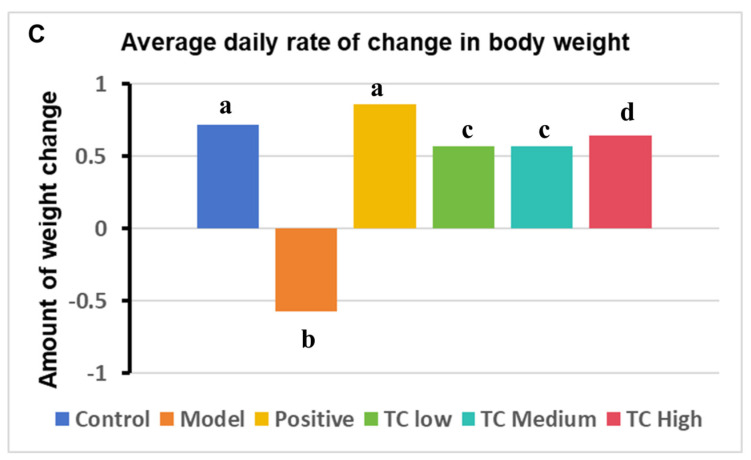
(**A**) Incidence of diarrhea in mice. (**B**) Weight changes in mice. (**C**) Average daily change in body weight. Groups with the same letter indicate no significant difference (*p* > 0.05), while groups with different letters indicate significant difference (*p* < 0.05).

Figure 1A shows that after successfully establishing an animal enteritis model, formal treatment experiments were conducted, and the diarrhea rate of each group of mice was calculated on day 14. The control, model, positive, TC low, TC medium, and TC high diarrhea rates were 0%, 83.3%, 20%, 50%, 41.7%, and 25%, respectively, indicating that TC has a certain protective effect against bacterial diarrhea in mice and is positively correlated with the dose.

Figure 1B shows that after the formal treatment trial, the weight loss in mice given TC via gavage improved and positively correlated with the dose.

The changes in the average daily body weight of the mice are shown in Figure 1C. After TC treatment, the body weight loss trend was altered and showed a positive correlation with dose compared with the attack model group; however, there was still a decrease in average daily body weight gain compared with the control group and the positive drug control group.

### 3.2. Pathological Changes in the Appendix and Stomach Tissues

**Figure 2:**
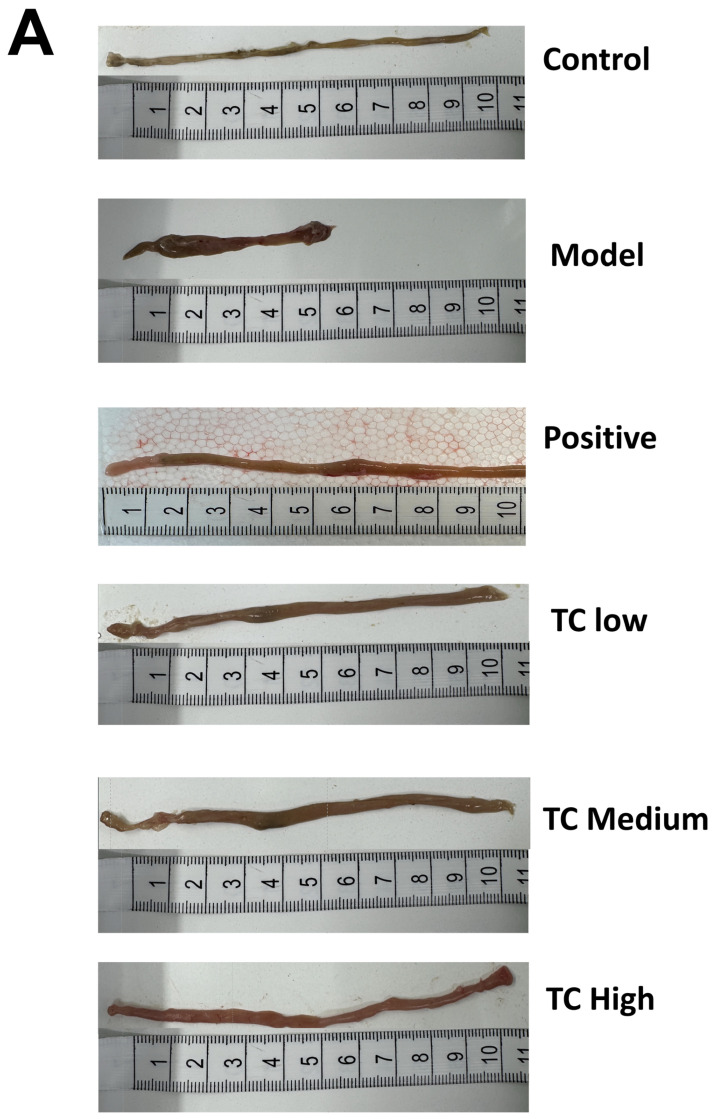

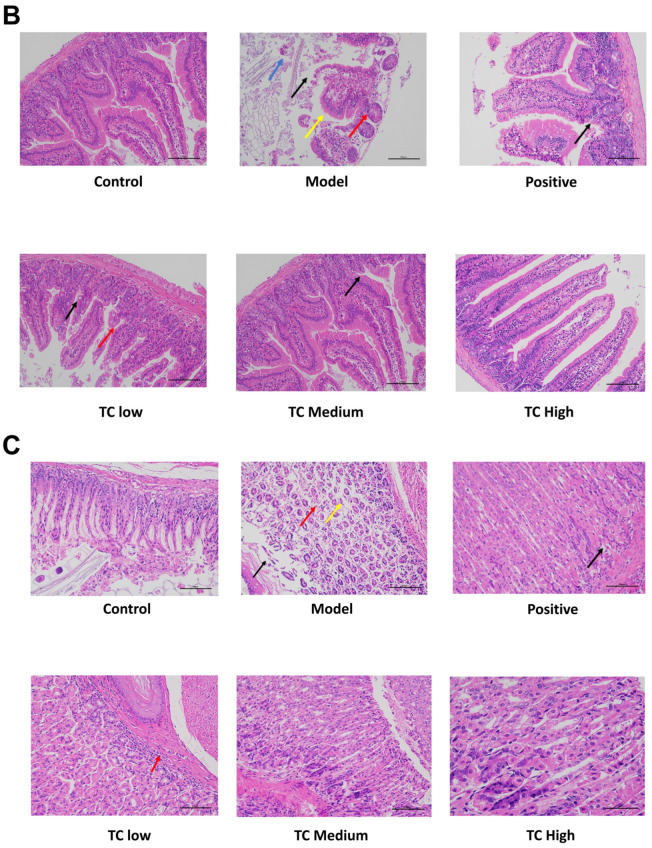
(**A**) Appearance of mouse small intestine. (**B**) Histological section of mouse cecal tissue. (**C**) Histological section of mouse stomach tissue.

Figure 2A shows morphological changes in the small intestines of the mice. In the model group, the tissue structure of the blind intestine was severely deformed, with ulceration, hemorrhage, hyperplasia resembling polyps, and numerous bleeding points. the TC low and TC medium groups exhibited mild symptoms of mucosal erosion and congestion. In the TC high group, the intestinal tissue had a normal morphology, but there was still some mild inflammatory reaction and congestion.

Figure 2B shows the histological section results of the cecum. No obvious pathological changes were found in the cecum tissue of the control group. In the model group, local ulceration was observed in the submucosal layer of the ileum tissue, with necrosis and sloughing of epithelial cells, dissolution of the intestinal glands in the substance layer, and the proliferation of connective tissue (black arrowhead). This was accompanied by a small amount of inflammatory lymphocyte infiltration (red arrowhead). Lymph nodes were hyperplastic in the subcutaneous layer (blue arrowhead), and partial atrophy of the gastric glands was observed in the substance layer, with a loose arrangement (yellow arrowhead). In the TC low and TC medium groups, only a small amount of inflammatory lymphocyte infiltration (black arrow) occurred in the cecal tissue. No obvious lesions were found in the TC high group.

Figure 2C shows the histological section of the stomach. In the control group, no obvious pathological changes were seen in the stomach tissue. In the model group, many cells were shed from the mucosal layer of the gastric tissue (black arrow), and there were loosely arranged local gastric gland atrophies in the subcutaneous layer (yellow arrow). There were also scattered inflammatory cells infiltrating the interstitial tissue (red arrow). In the TC low group, only a small amount of cell shedding (red arrow) was visible in the submucosal layer of the gastric tissue. There were no obvious pathological changes in the TC medium and TC high groups.

### 3.3. Results of In Vivo Safety Assessment

**Figure 3:**
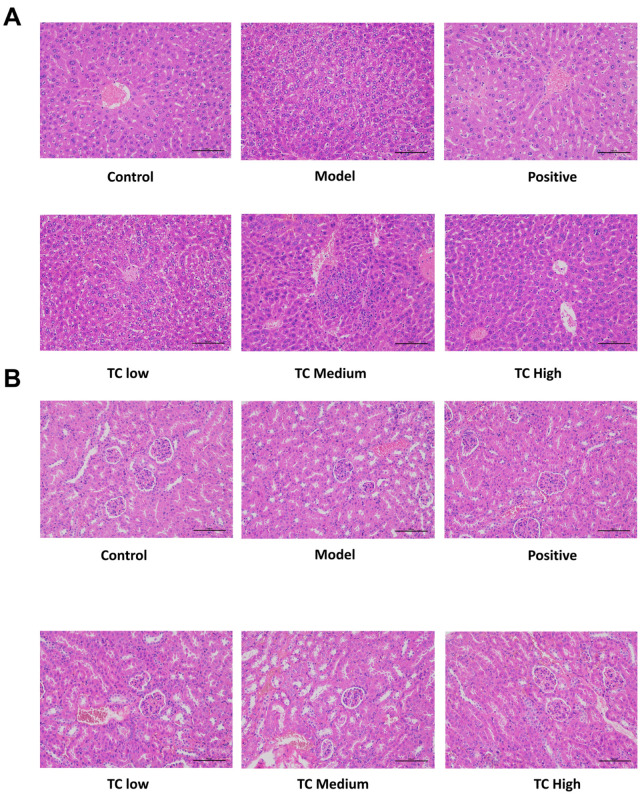
(**A**) Pathological changes in mouse liver. (**B**) Pathological changes in mouse kidneys.

The HE staining results for the liver and kidneys are shown in Figure 3. Histological examination did not reveal any adverse effects from TC administration on the kidneys or liver. No pathological changes were found in the liver and kidney tissue sections of the mice in each group.

### 3.4. Inflammatory and Anti-Inflammatory Factor Detection

Serological parameters are usually more intuitive indicators of the extent of cell damage in the body, and pro-inflammatory and anti-inflammatory factors serve as dynamic monitoring indicators. Dynamic changes in the levels of these factors are closely related to the severity of disease and inflammation in the body [22]. Therefore, we studied the serum biochemical parameters of each group. The results show that TC has a certain anti-inflammatory effect.

**Figure 4:**
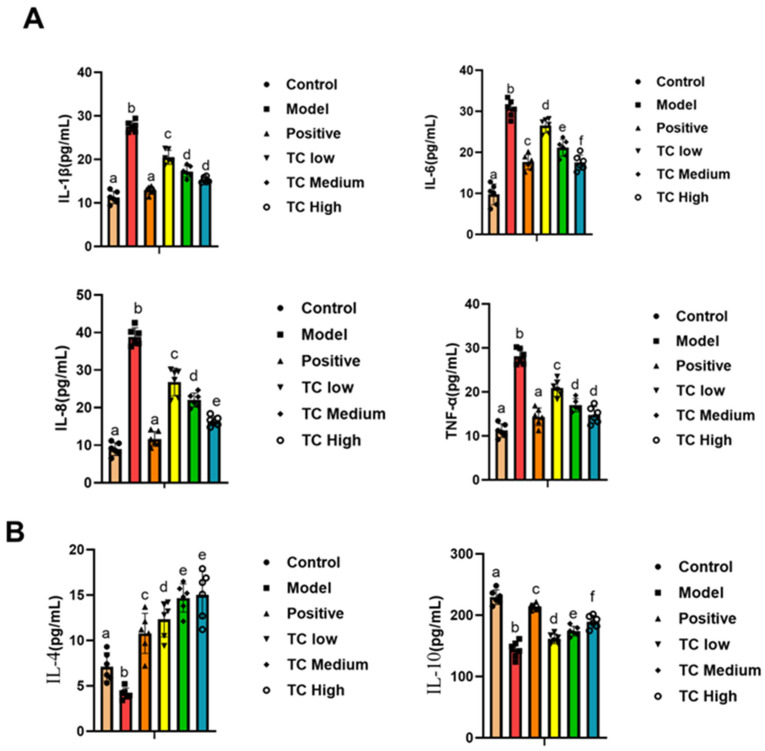
(**A**) Determination of pro-inflammatory factors in mouse serum. (**B**) Determination of anti-inflammatory factors in mouse serum. Lowercase letters that differ represent a significant difference at *p* < 0.05.

As shown in Figure 4, compared with the control group, the IL-1β, IL-6, IL-8, and TNF-α levels were significantly increased (*p* < 0.05) in the model group, while the IL-10 and IL-4 levels were significantly decreased (*p* < 0.05).

Compared with the model group, the TC treatment group showed a significant decrease in IL-1β, IL-6, IL-8, and TNF-α (*p* < 0.05) and a significant increase in IL-10 and IL-4 (*p* < 0.05). The changes in pro-inflammatory cytokines and anti-inflammatory cytokines were positively correlated with the TC concentration used in the treatment. The results indicate that TC can reduce inflammatory responses in mice and promote recovery.

### 3.5. Determination of Antioxidant Indicators in Mouse Serum

**Figure 5:**
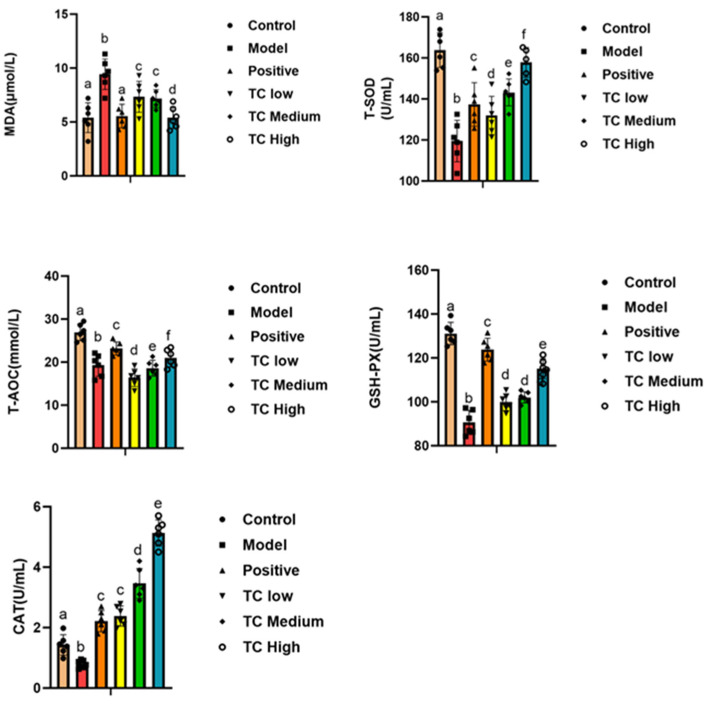
Determination of antioxidant indicators in mouse serum. Lowercase letters that differ represent a significant difference with *p* < 0.05.

By detecting antioxidant markers, one can reveal changes in antioxidant capacity during the course of a disease, providing clues for the study of its pathogenesis. We studied the serum antioxidant indicators in each group. Figure 5 shows that compared with the control group, MDA in the model group was significantly increased (*p* < 0.05), while T-SOD, T-AOC, GSH-PX, and CAT were significantly decreased (*p* < 0.05). Compared with the model group, the TC group showed a significant decrease in MDA (*p* < 0.05) and a significant increase in T-SOD, T-AOC, GSH-PX, and CAT (*p* < 0.05). Furthermore, the TC high group was closer to the positive group, and the changes in antioxidant indicators in the TC treatment group were positively correlated with the TC concentration. The results show that TC can reduce the degree of disease damage in mice and enhance their antioxidant abilities.

### 3.6. 16S rDNA Metagenomic Analysis of Gut Contents in Mice

**Figure 6:**
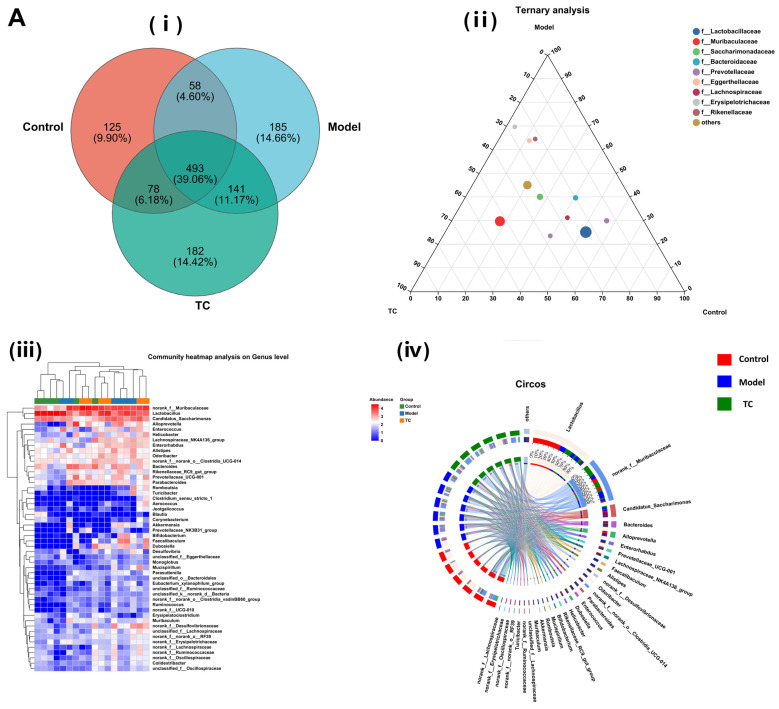

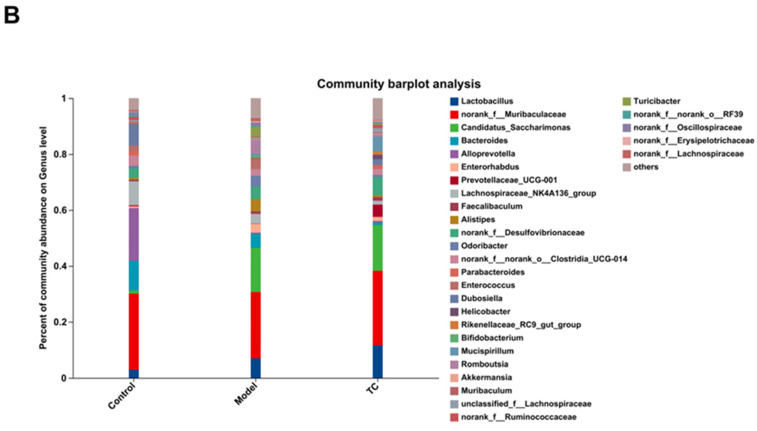

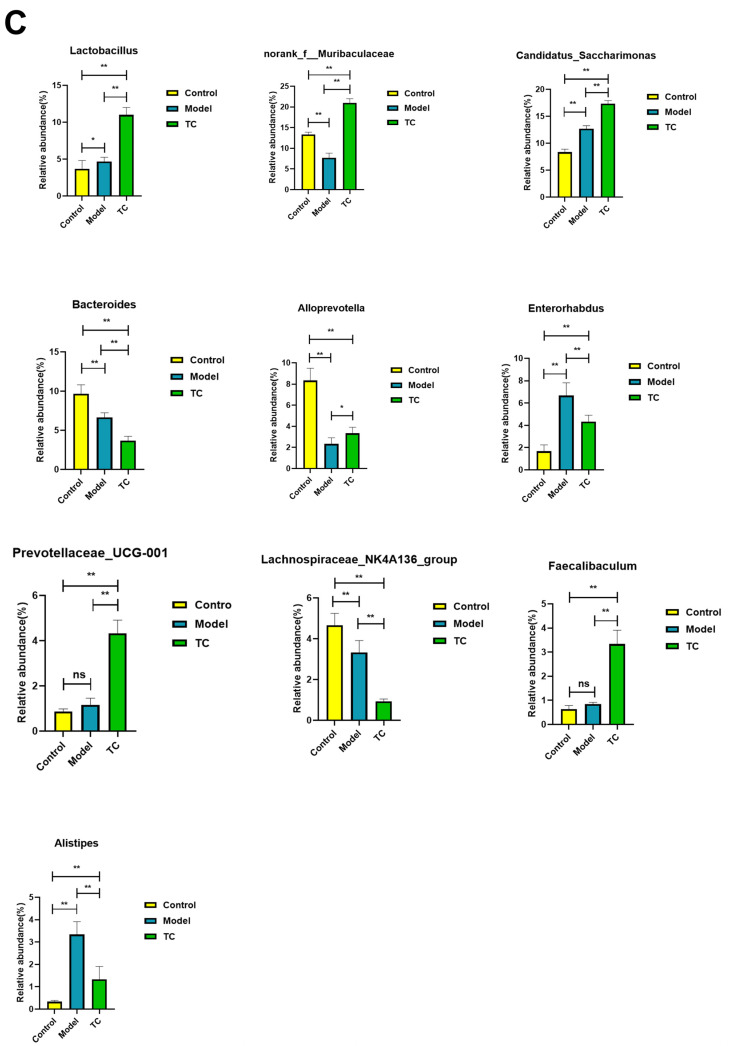
The effect of TC on the gut microbiome composition of bacterial diarrhea mice. (**A**) (**i**) Venn diagrams for different groups; (**ii**) different groups of Ternary triangular diagrams; (**iii**) a heatmap chart showing different groups of bacterial colonies; (**iv**) Circos sample and species relationship map. (**B**) Histogram of relative abundance of dominant bacterial populations in different groups. (**C**) Differences in major advantages of bacterial colony analysis between different groups. “ns”represents no significant difference; “*” represents a significant difference (*p* < 0.05); “**” represents an extremely significant difference (*p* < 0.01).

Figure 6A(i) shows the following results: The control, model, and TC treatment groups had 754, 877, and 894 operational taxonomic units (OTUs), respectively, in the intestinal microbiome, indicating that TC can alter the composition of the mouse intestinal microbiome. At the same time, TC can alter the structure of the intestinal microbiome of mice with *Salmonella*-induced enteritis. Figure 6A(ii) shows the following results: *f__Lactobacillaceae*, *f__Muribaculaceae*, *f__Saccharimonadaceae*, *f__Bacteroidaceae*, *f__Prevotellaceae*, *f__Eggerthellaceae*, *f__Lachnospiraceae*, *f__Erysipelotrichaceae*, and *f__Rikenellaceae* are the top 10 species in terms of average relative abundance among the bacterial communities associated with *Salmonella* pathogenesis and TC treatment. These bacterial communities may play a key role in the relevant functional pathways. Further analysis of the changes in gut-specific microbial communities at the genus level was conducted for the control, model, and TC treatment groups. The results can be seen in Figure 6A(iii),(iv): The TC treatment group mainly increased the relative abundance of *norank_f__Muribaculaceae*, *Lactobacillus*, *Candidatus_Saccharimonas*, *Alloprevotella*, *Enterococcus*, *Helicobacter*, *Lachnospiraceae_NK4A136_group*, *Enterorhabdus*, *Alistipes*, *Odoribacter*, *norank_f__norank_o__Clostridia_UCG-014*, *Bacteroides*, *Rikenellaceae_RC9_gut_group*, and *Prevotellaceae_UCG-001*, while *Salmonella* infection decreased the abundance of these microbial communities.

*Salmonella* infection increased the relative abundance of *Romboutsia*, *Turicibacter*, *Clostridium_sensu_stricto_1*, *Aerococcus*, *Jeotgalicoccus*, *Blautia*, *Corynebacterium*, *Akkermansia*, *Prevotellaceae_NK3B31_group*, *Bifidobacterium*, *Faecalibaculum*, *Dubosiella*, *Desulfovibrio*, *unclassified_f__Eggerthellaceae*, *Monoglobus*, *Mucispirillum*, *Parasutterella*, *unclassified_o__Bacteroidales*, *Eubacterium_xylanophilum_group*, *unclassified_f__Ruminococcaceae*, *unclassified_k__norank_d__Bacteria*, *norank_f__norank_o__Clostridia_vadinBB60_group*, *Ruminococcus*, *norank_f__UCG-010*, *Erysipelatoclostridium*, *Muribaculum*, *norank_f__Desulfovibrionaceae*, *unclassified_f__Lachnospiraceae*, *norank_f__norank_o__RF39*, *norank_f__Erysipelotrichaceae*, *norank_f__Lachnospiraceae*, *norank_f__Ruminococcaceae*, *norank_f__Oscillospiraceae*, *Colidextribacter*, and *unclassified_f__Oscillospiraceae*. The above statistical results indicate that *Salmonella* infection can significantly change the structure of the intestinal microbiome. The relative abundance of *norank_f__Muribaculaceae*, *Lactobacillus*, *Candidatus_Saccharimonas*, *Alloprevotella*, *Enterococcus*, *Helicobacter*, *Lachnospiraceae_NK4A136_group*, *Enterorhabdus*, *Alistipes*, *Odoribacter*, *norank_f__norank_o__Clostridia_UCG-014*, *Bacteroides*, *Rikenellaceae_RC9_gut_group*, and *Prevotellaceae_UCG-001* can be restored with TC. These results indicate that TC can effectively restore the reduced abundance of some species in the gut microbiome caused by *Salmonella* infection, with *Lactobacillus*, *norank_f__Muribaculaceae*, *Candidatus_Saccharimonas*, *Bacteroides*, *Alloprevotella*, *Enterorhabdus*, *Prevotellaceae_UCG-001*, *Lachnospiraceae_NK4A136_group*, *Faecalibaculum*, *Alistipes*, and *norank_f__Desulfovibrionaceae* potentially being the core functional bacterial communities responsible for its antibacterial activity against *Salmonella* infection.

Figure 6B shows the following results: In the control, model, and TC treatment groups, the core bacterial communities showing major changes were *Lactobacillus*, *norank_f__Muribaculaceae*, *Candidatus_Saccharimonas*, *Bacteroides*, *Alloprevotella*, *Enterorhabdus*, *Prevotellaceae_UCG-001*, *Lachnospiraceae_NK4A136_group*, *Faecalibaculum*, *Alistipes*, *norank_f__Desulfovibrionaceae*, *Odoribacter*, *norank_f__norank_o__Clostridia_UCG-014*, *Parabacteroides*, *Enterococcus*, *Dubosiella*, *Helicobacter*, *Rikenellaceae_RC9_gut_group*, *Bifidobacterium*, *Mucispirillum*, *Romboutsia*, *Akkermansia*, *Muribaculum*, *unclassified_f__Lachnospiraceae*, *norank_f__Ruminococcaceae*, *Turicibacter*, *norank_f__norank_o__RF39*, *norank_f__Oscillospiraceae*, *norank_f__Erysipelotrichacea*, and *norank_f__Lachnospiraceae*. Meanwhile, the top 10 bacterial communities in terms of differential abundance were selected for a significance analysis, as shown in Figure 6C. Compared with the control group, in the model group the abundance of the *Lactobacillus* bacterial community (*p* < 0.05) and the *Candidatus_Saccharimonas*, *Enterorhabdus*, and *Alistipes* bacterial communities (*p* < 0.01) significantly increased, and the abundance of the *norank_f__Muribaculaceae*, *Bacteroides*, *Alloprevotella*, and *Lachnospiraceae_NK4A136_group* bacterial communities (*p* < 0.01) significantly decreased. Compared with the model group, the TC group showed a significant recovery effect in the *Alloprevotella* community (*p* < 0.05) and an extremely significant recovery effect in the *norank_f__Desulfovibrionaceae* and *Faecalibaculum* communities (*p* < 0.01). There was highly significant attenuation (*p* < 0.01) in the abundance of the *Enterorhabdus* and *Alistipes* bacterial groups caused by *Salmonella*, but the abundance of the *Lactobacillus* and *Candidatus_Saccharimonas* bacterial groups still significantly increased (*p* < 0.01). For *Bacteroides* and *Lachnospiraceae_NK4A136_group*, there was still a highly significant downward trend (*p* < 0.01). For the TC group, compared with the control and model groups, *Prevotellaceae_UCG-001* and *Faecalibaculum* showed highly significant upward trends (*p* < 0.01).

### 3.7. Mouse Intestinal Content ITS Microbial Sequencing and Data Analysis

**Figure 7:**
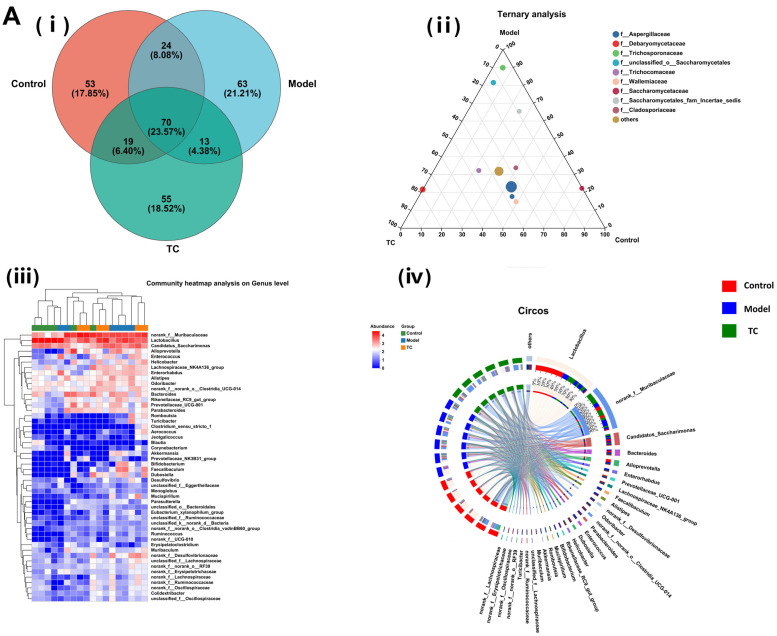

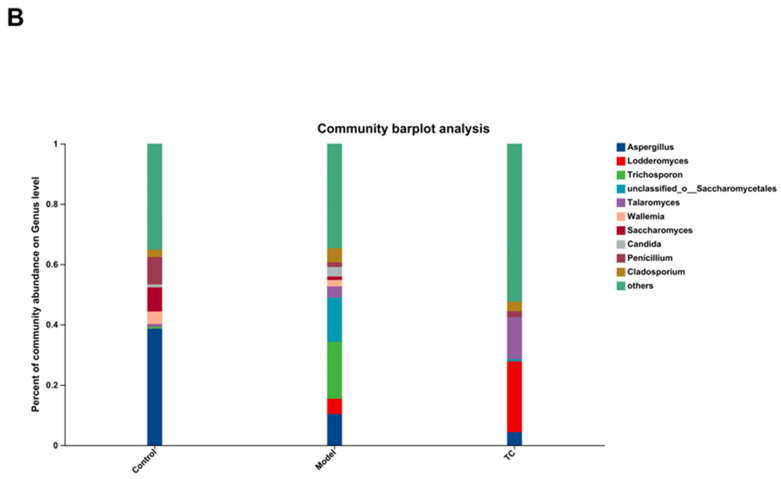

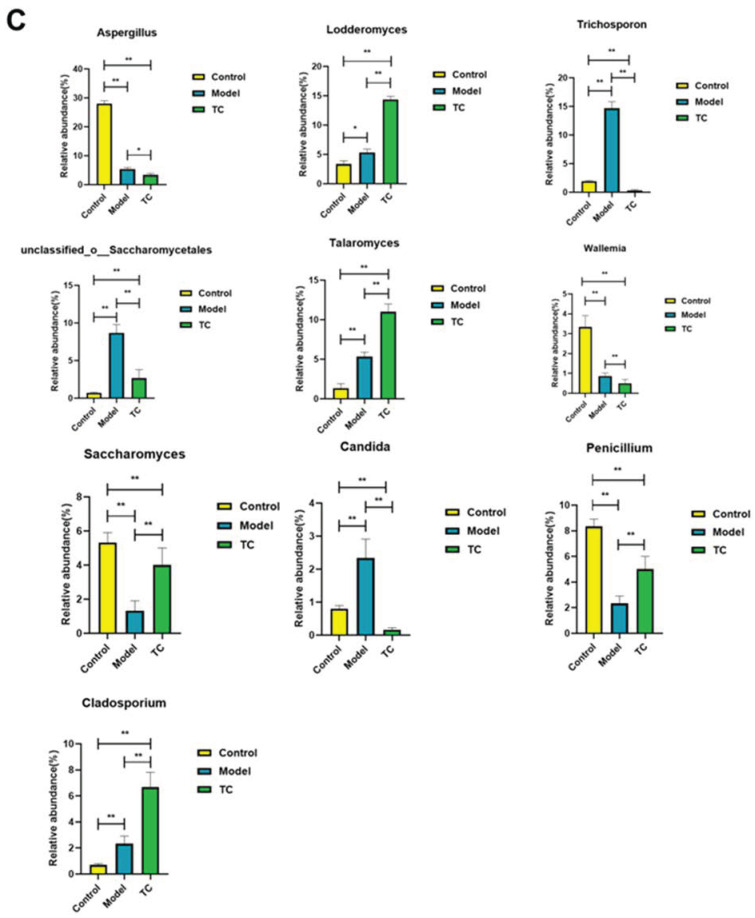
Effect of TC on the composition of ITS gut microbiome in bacterial diarrhea mice. (**A**) (**i**) Venn diagrams for different groups; (**ii**) different groups of Ternary triangular diagrams; **(iii)** a heatmap chart showing different groups of bacterial colonies; (**iv**) Circos sample and species relationship map. (**B**) Histogram of relative abundance of dominant bacterial populations in different groups. (**C**) Differences in major advantages of bacterial colony analysis among different groups. “*” represents a significant difference (*p* < 0.05); “**” represents an extremely significant difference (*p* < 0.01).

Figure 7A(i) shows the following results: The control, model, and TC treatment groups had 166, 170, and 156 intestinal microbial operational taxonomic units (OTUs), respectively, indicating that TC can alter the composition of the mouse gut fungal community.

Figure 7A(ii) shows the following results: *f__Aspergillaceae*, *f__Debaryomycetaceae*, *f__Trichosporonaceae*, *f__unclassified_o__Saccharomycetales*, *f__Trichocomaceae*, *f__Wallemiaceae*, *f__Saccharomycetaceae*, *f__Saccharomycetales_fam_Incertae_sedis*, and *f__Cladosporiaceae* are among the top 10 fungal communities that contribute to *Salmonella* pathogenesis and TC treatment; these fungal communities may play a critical role in the relevant functional pathways. Further analysis of the changes in specific intestinal fungal communities at the genus level was conducted for the control, model, and TC treatment groups. The results are shown in Figure 7A(iii),(iv). In the TC treatment group, the relative abundance of fungal communities, such as *Aspergillus*, *Trichomonascus*, *unclassified_o__Saccharomycetales*, *unclassified_k__Fungi*, *Penicillium*, *Talaromyces*, *Cladosporium*, *Candida*, *Wallemia*, *Filobasidium*, *Alternaria*, *Lodderomyces*, *Trichosporon*, *Fusarium*, *Trichoderma*, *Saccharomyces*, *Papiliotrema*, and *Cutaneotrichosporon*, mainly increased, whereas *Salmonella* infection decreased the abundance of these fungal communities. *Salmonella* infection increased the relative abundance of *Sarocladium*, *Microascus*, *Sporidiobolus*, *Byssochlamys*, *unclassified_o__Eurotiales*, *Saccharomycopsis*, *Didymella*, *Naganishia*, *Debaryomyces*, *Bullera*, *Epicoccum*, *Cystofilobasidium*, *Wickerhamomyces*, *Ilyonectria*, *Issatchenkia*, *Cephalotrichum*, *Wardomyces*, *Trichocladium*, *Rhodotorula*, *Mortierella*, *Pseudogymnoascus*, *unclassified_f__Nectriaceae*, *Xeromyces*, *Symmetrospora*, *Cystobasidium*, *unclassified_p__Ascomycota*, *Holtermanniella*, *Apiotrichum*, *Vishniacozyma*, *Udeniomyces*, *Thermomyces*, and *Acremonium*.

These statistical results indicate that *Salmonella* infection can significantly change the structure of the intestinal fungal microbiome. The relative abundance of *Aspergillus*, *Trichomonascus*, *unclassified_o__Saccharomycetales*, *unclassified_k__Fungi*, *Penicillium*, *Talaromyces*, *Cladosporium*, *Candida*, *Wallemia*, *Filobasidium*, *Alternaria*, *Lodderomyces*, *Trichosporon*, *Fusarium*, *Trichoderma*, *Saccharomyces*, *Papiliotrema*, and *Cutaneotrichosporon* downregulated by *Salmonella*-induced enteritis can be restored with TC. These results suggest that TC can effectively restore the abundance of intestinal microbiota in certain fungal species altered by *Salmonella* infection, including *Aspergillus*, *Lodderomyces*, *Trichosporon*, *unclassified_o__Saccharomycetales*, *Talaromyces*, *Wallemia*, *Saccharomyces*, *Candida*, *Penicillium*, *Cladosporium*, *Filobasidium*, *Cutaneotrichosporon*, *Fusarium*, *unclassified_k__Fungi*, *Alternaria*, *Trichomonascus*, *Saccharomycopsis*, *Thermomyces*, *Sarocladium*, *Apiotrichum*, *Papiliotrema*, *Byssochlamys*, *Cystofilobasidium*, *Acremonium*, *unclassified_p__Ascomycota*, *Vishniacozyma*, *Microascus*, *Didymella*, and *Trichoderma*, *unclassified_o__Eurotiales*. These may be the core functional fungal communities contributing to the antibacterial activity of TC against *Salmonella* infection.

Figure 7B,C show the following results: *Aspergillus*, *Lodderomyces*, *Trichosporon*, *unclassified_o__Saccharomycetales*, *Talaromyces*, *Wallemia*, *Saccharomyces*, *Candida*, *Penicillium*, and *Cladosporium* were the main differential core fungal communities in each group. The top 10 most abundant differential fungal communities were selected for significance analysis. Compared with the control group, in the model group the *Lodderomyces* fungal community(*p* < 0.05) and the abundance of the *Trichosporon*, *unclassified_o__Saccharomycetales*, *Talaromyces*, *Candida*, and *Cladosporium* fungal communities (*p* < 0.01) significantly increased; conversely, the *Aspergillus*, *Wallemia*, *Saccharomyces*, and *Penicillium* fungal community abundances significantly decreased (*p* < 0.01). Compared with the model group, the TC group demonstrated an extremely significant recovery effect in the *Saccharomyces* and *Penicillium* fungal communities (*p* < 0.01) and in the *Trichosporon*, *unclassified_o__Saccharomycetales*, and *Candida* fungal communities (*p* < 0.01). The abundance of the *Aspergillus* fungal community showed a significant decrease (*p* < 0.05), and the abundance of the *Wallemia* fungal community showed a highly significant decrease (*p* < 0.01). The abundance of the *Lodderomyces*, *Talaromyces*, and *Cladosporium* fungal communities showed highly significant upward or downward trends (*p* < 0.01).

### 3.8. Metabolite Profiling Analysis for Each Group

**Figure 8:**
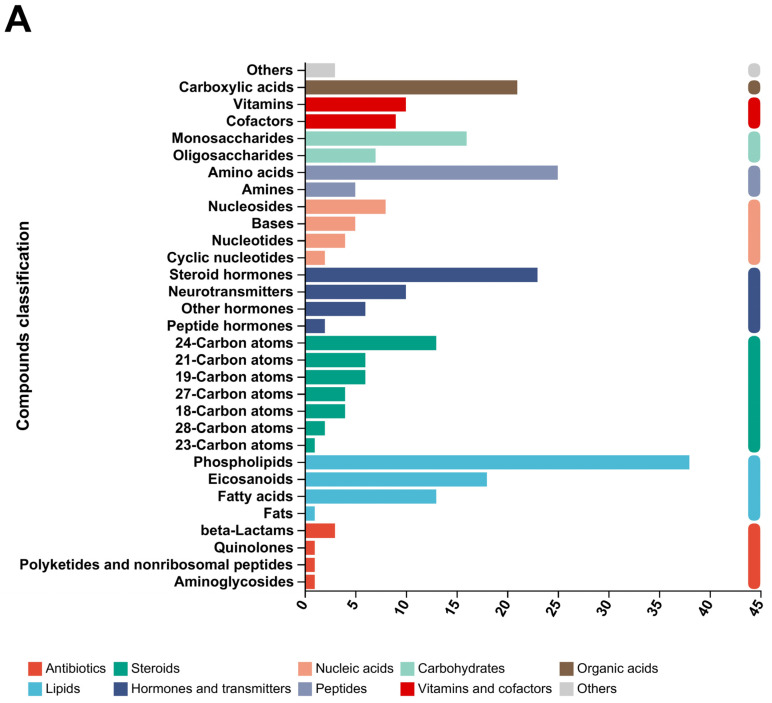

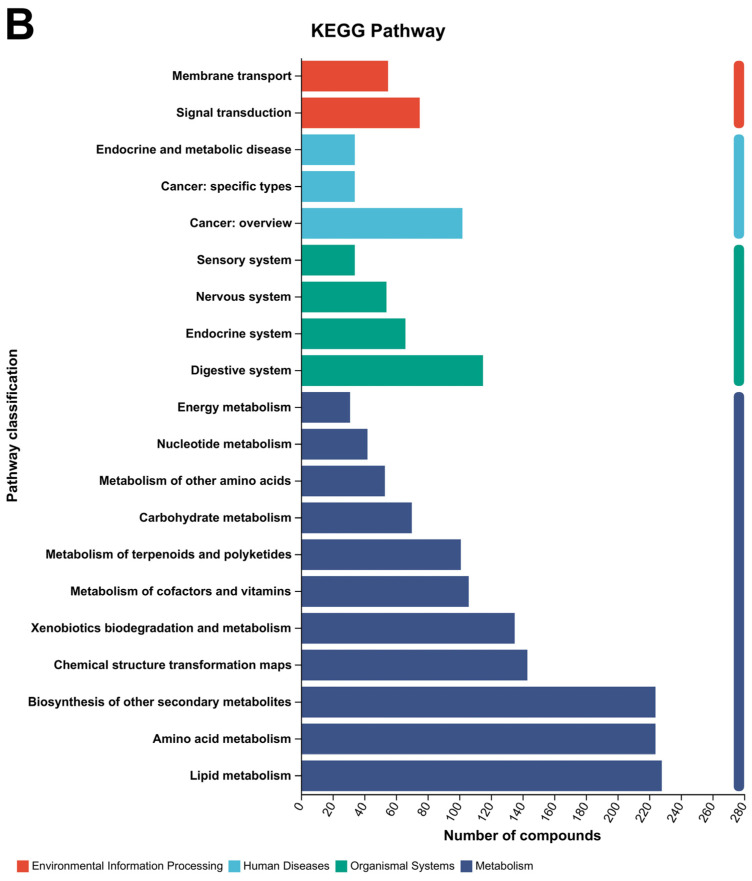

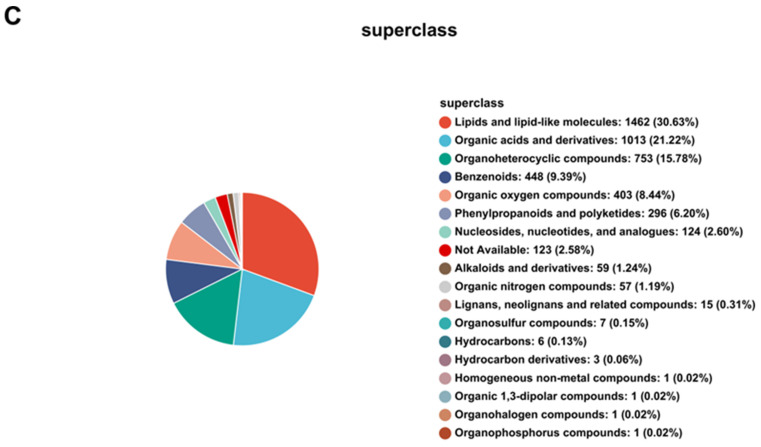
Principal metabolite function enrichment analysis by group. (**A**) Differential metabolite profiling analysis. (**B**) Metabolic pathways of differential metabolites. (**C**) Major metabolite HMDB compound classification.

Through KEGG enrichment analysis, the main differences in metabolites between the groups were analyzed, as shown in Figure 8A. The main differential metabolites are as follows: Organic acids (carboxylic acids), Vitamins and cofactors (Vitamins, Cofactors), Carbohydrates (Monosaccharides, Oligosaccharides), Hormones and transmitters (Steroid hormones, Neurotransmitters, Other hormones, Peptide hormones), Steroids (24-Carbon atoms, 21-Carbon atoms, 19-Carbon atoms, 27-Carbon atoms, 18-Carbon atoms, 28-Carbon atoms, 23-Carbon atoms), Lipids (Phospholipids, Eicosanoids, Fatty acids, Fats), and Antibiotics (beta-Lactams, Quinolones, Polyketides and non-ribosomal peptides, Aminoglycosides).

The metabolic pathways of the main differential metabolites are as follows (Figure 8B): Environmental Information Processing (Membrane transport, Signal transduction); Human Diseases (Endocrine and metabolic disease, Cancer: specific types, Cancer: overview); Organismal Systems (Sensory system, Nervous system, Endocrine system, Digestive system); Metabolism (Energy metabolism, Nucleotide metabolism, Metabolism of other amino acids, Carbohydrate metabolism, Metabolism of terpenoids and polyketides, Metabolism of cofactors and vitamins, Xenobiotics biodegradation and metabolism, Chemical structure transformation maps, Biosynthesis of other secondary metabolites, Amino acid metabolism).

The main metabolites of each group were classified into HMDB compounds and statistically analyzed, as shown in Figure 8C. The top 10 HMDB compounds were as follows: Lipids and lipid-like molecules: 1462 (30.63%); Organic acids and derivatives: 1013 (21.22%); Organoheterocyclic compounds: 753 (15.78%); Benzenoids: 448 (9.39%); Organic oxygen compounds: 403 (8.44%); Phenylpropanoids and polyketides: 296 (6.20%); Nucleosides, nucleotides, and analogues: 124 (2.60%); Not available: 123 (2.58%); Alkaloids and derivatives: 59 (1.24%); Organic nitrogen compounds: 57 (1.19%).

**Figure 9:**
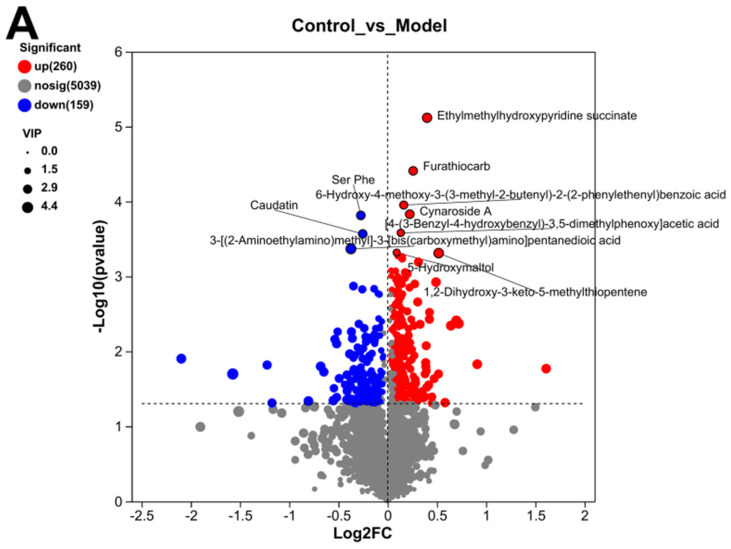

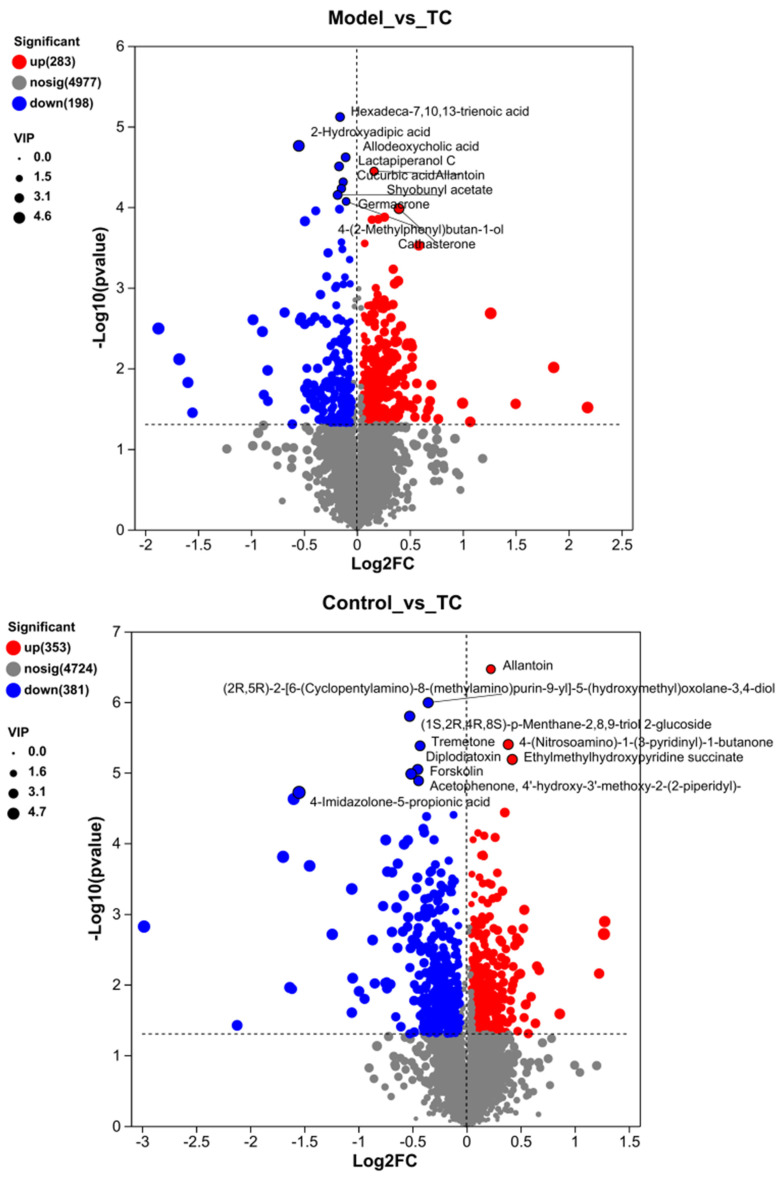

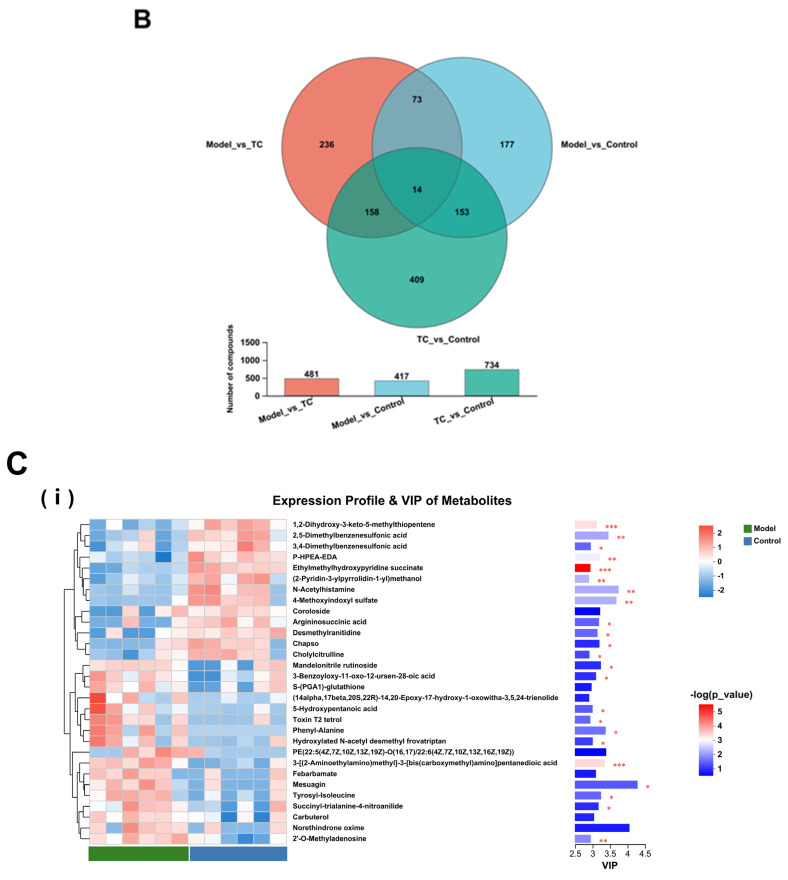

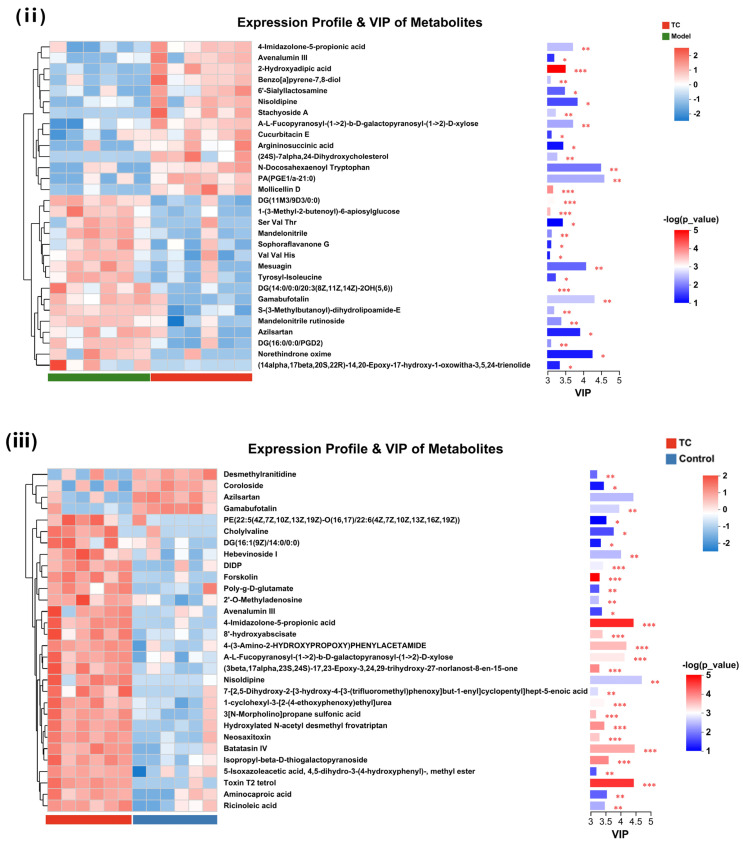

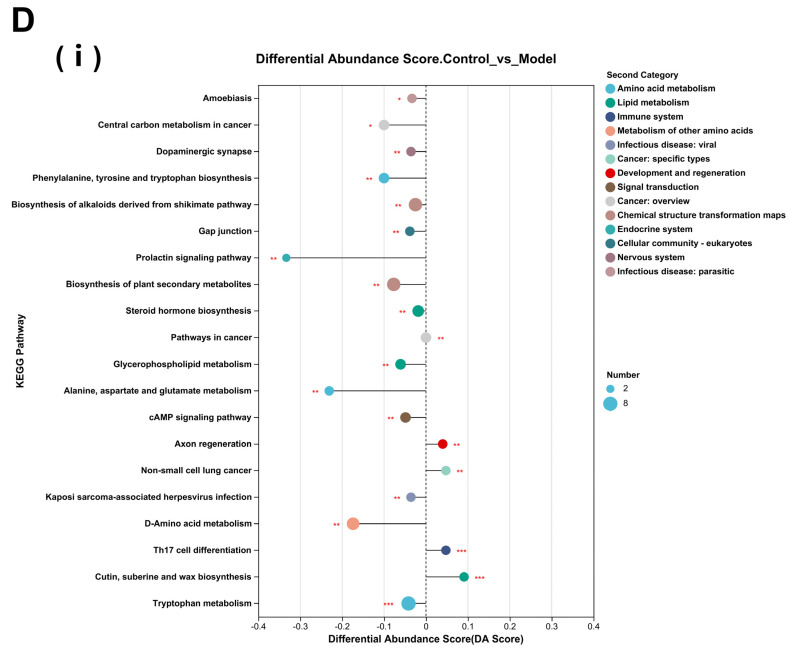

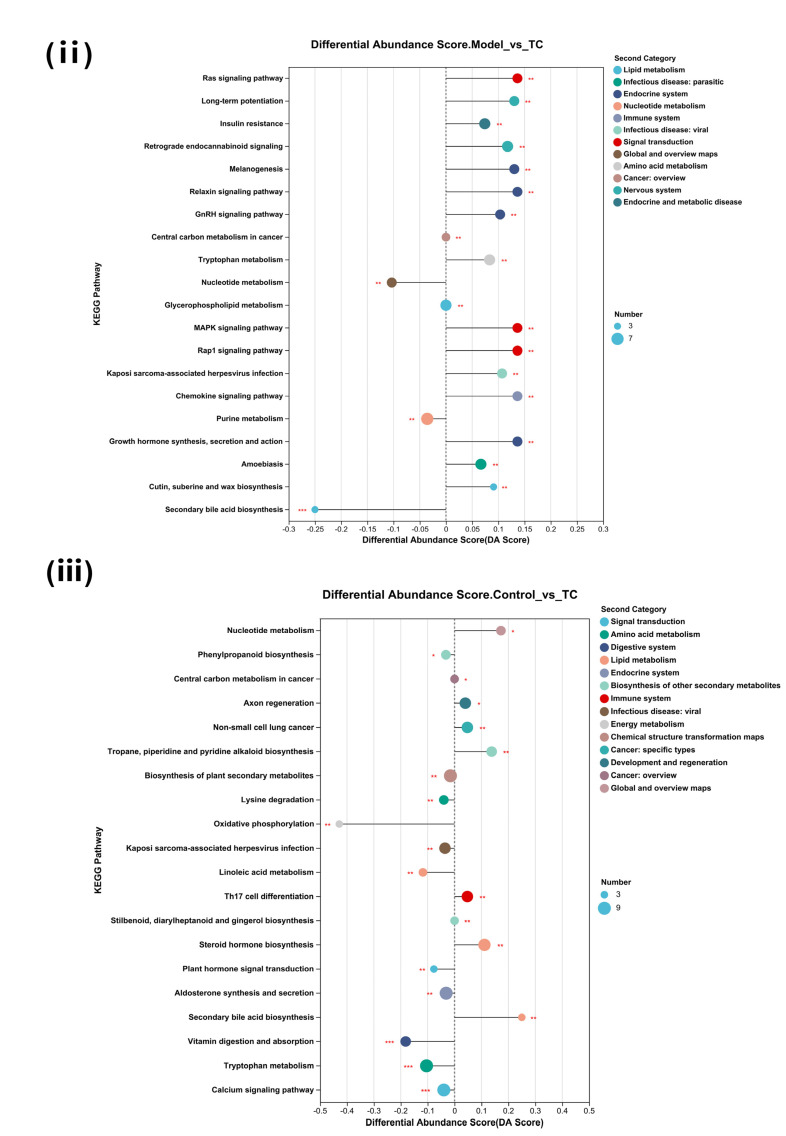
Differential metabolite analysis between different groups. (**A**) Analysis of volcanic ion charts between different groups. (**B**) Venn diagram of metabolites across different groups. (**C**) Heatmap clustering diagram of differential metabolites in different groups and significant analysis of differential metabolites: (**i**) model group vs. TC group; (**ii**) model group vs. TC group; (**iii**) control group vs. TC group. (**D**) Significance analysis of KEGG pathway enrichment for differential metabolites across different groups: (**i**) model group vs. TC group; (**ii**) model group vs. TC group; (**iii**) control group vs. TC group. The horizontal axis in the figure represents the difference abundance score (DA Score), and the vertical axis represents the length of the line segment for the KEGG metabolic pathway name, which represents the absolute value of the DA Score. The size of the dots represents the number of annotated differential metabolites in the pathway, with larger dots indicating more differential metabolites in the pathway. The distribution of dots on the right side of the central axis and the longer the line segment indicate that the overall expression of the pathway tends to be upregulated. The distribution of dots on the left side of the central axis and the longer the line segment indicate that the overall expression of the pathway tends to be downregulated. “*” represents *p* < 0.05, “**” represents *p* < 0.01, and “***” represents *p* < 0.001.

The specific metabolites with significant differences between the control group, model group, and TC group were analyzed. A volcano plot of positive and negative ions between the groups was analyzed, and the results can be seen in Figure 9A, Compared with the control group, the model group mainly upregulated Ethylmethylhydroxypyridine succinate (−Log10(*p*-value):5.1177031990623485), Furathiocarb (−Log10(*p*-value):4.408824049688208), and Cynaroside A (−Log10(*p*-value):3.8303255659411932). The main downregulated proteins were Ser Phe (−Log10 (*p*-value):31814174640387038) and Caudatin (−Log10(*p*-value):35676722077383958).

Compared with the model group, the TC group mainly showed an upregulation of Allantoin (−Log10(*p*-value):4.443697499232712) and Cathasterone (−Log10(*p*-value):3.9800533183211577). The main downregulated proteins were Hexadeca-7.10.13-trienoic acid (−Log10(*p*-value):5.117418546445549), Allodeoxyehotic acid (−Log10(*p*-value):4.617442678091214), Cucurbic acid (−Log10(*p*-value):4.311135431945208), and Germacrone (−Log10(*p*-value):4.229663558904851).

A Venn diagram of metabolites between the control group, model group, and TC group is shown in Figure 9B. The metabolites in the intersections of the control group and model group, the model group and TC group, and the control group and TC group numbered 481, 417, and 734, respectively. A large number of overlapping compounds indicate that *Salmonella* infections and TC extract treatments can lead to dramatic changes in the metabolites of an organism, indirectly disrupting and restoring the internal environment and system. There were 14 metabolites in common among the three groups, which may be the core metabolites involved in the pathogenesis of *Salmonella* and the therapeutic effects of TC.

A significant difference analysis was conducted on the differential metabolites between the control group, model group, and TC group according to their correlations. The results are shown in Figure 9C. Among the listed metabolites, 80% (24/30) of those between the model group and control group were significantly different (*p* < 0.05), including 1,2-Dihydroxy-3-keto-5-methylthiopentene, Ethylmethylhydroxypyridine succinate, and 3-[(2-Aminoethylamino)methyl]-3-[bis(carboxymethyl)amino]pentanedioic acid, which was extremely significant (*p* < 0.001). Mesuagin and Norethindrone oxime had higher contribution values in the upregulated metabolites, while N-Acetylhistamine, 4-Methoxyindoxyl sulfate, and 2,5-Dimethylbenzenesulfonic had higher contribution values in the downregulated metabolites. Between the model group and the TC group, all the listed metabolites showed significant differences (*p* < 0.05), among which, 2-Hydroxyadipic acid, Mollicellin D, DG (11M3/9D3/0:0), 1-(3-Methyl-2-butenoyl)-6-apiosylglucose, and DG (14:0/0:0/20:3(8Z,11Z,14Z)-2OH(5,6)) showed extremely significant differences (*p* < 0.001). Gamabufotalin, Norethindrone oxime, Mesuagin, and Azilsartan had high contribution values in the upregulated metabolites, while PA (PGE1/a-21:0), N-Docosahexaenoyl Tryptophan, Nisoldipine, and A-L-Fucopyranosyl-((1- > 2))-b-D-galactopyranosyl-((1- > 2))-D-xylose had high contribution values in the downregulated metabolites. In the TC group, all 30 metabolites listed showed significant differences (*p* < 0.05) compared with the control group. Of these, DIDP, Forskolin, 4-Imidazolone-5-propionic acid, 8′-hydroxyabscisate, 4-(3-Amino-2-HYDROXYPROPOXY)PHENYLACETAMIDE, A-L-Fucopyranosyl-((1- > 2))-b-D-galactopyranosyl-((1- > 2))-D-xylose, (3beta,17alpha,23S,24S)-17,23-Epoxy-3,24,29-trihydroxy-27-norlanost-8-en-15-one, 1-cyclohexyl-3-[2-(4-ethoxyphenoxy)ethyl]urea, 3[N-Morpholino]propane sulfonic acid, Hydroxylated N-acetyl desmethyl frovatriptan, Neosaxitoxin, Batatasin IV, Isopropyl-beta-D-thiogalactopyranoside, and Toxin T2 tetrol showed extremely significant differences (*p* < 0.001). Nisoldipine, Batatasin IV, Toxin T2 tetrol, and 4-Imidazolone-5-propionic acid had higher contribution values in the upregulated metabolites, while Azilsartan and Gamabufotalin had higher contribution values in the downregulated metabolites.

The differential metabolites were subjected to KEGG pathway enrichment analysis for each group, and most of the metabolic pathways showed a downregulated inhibitory trend compared with the control group (16/20). *Salmonella* downregulated the body’s metabolism and circulatory system. There was a marked upregulation of axon regeneration and non-small cell lung cancer (*p* < 0.01) and a highly significant upregulation of Th17 cell differentiation, cutin, suberine, and wax biosynthesis processes (*p* < 0.001). There was also a significant downregulation of the prolactin signaling pathway, alanine, aspartate and glutamate metabolism, and D-amino acid metabolism (*p* < 0.01). The TC group showed an upregulation of most of the metabolic processes and pathways compared with the model group, indicating that the entire body’s circulatory system and metabolic processes improved. There was a marked increase in the Ras signaling pathway, long-term potentiation, the MAPK signaling pathway, the Rap1 signaling pathway, the chemokine signaling pathway, and growth hormone synthesis, secretion, and action (*p* < 0.01). There was a marked decrease in the nucleotide metabolism and purine metabolism processes (*p* < 0.01) and an extremely significant decrease in the secondary bile acid biosynthesis process (*p* < 0.001).

## 4. Discussion

*Salmonella* not only causes diseases in animals but also poses a serious threat to food safety and is closely related to human health [23]. The yak is the main economic livestock species for farmers and herders in the Qinghai–Tibet Plateau area and is a common source of meat, milk, wool, and leather for local herders [24]. However, the negative impact of *Salmonella* infection on livestock farming has been worsening, with the pathogen causing diarrhea and even death, resulting in huge economic losses for local farmers and herders [25]. Antibiotics are inevitably used for the clinical treatment of *Salmonella* and have become one of the most frequently used methods [26]. However, frequent use can lead to the formation of multi-resistant bacteria, which will become a significant challenge in food and biological safety [27]. Under the unified principle of “One Health”, antibiotic resistance (ABR) is a growing public health concern worldwide [28]. In order to prevent the formation of bacterial antibiotic resistance, selecting and researching antibiotic alternatives have become common research topics. Among the many alternative antibiotic solutions, natural plant extracts have become a new focus, as they have the advantages of easy acquisition, wide availability, safety and effectiveness, and naturalness [29]. Based on the above research background, this experiment selected TC—a natural plant medicine widely grown in the Tibetan Plateau region—as the research object and prepared its aqueous extract. This study investigated the therapeutic effects of TC on mouse gastroenteritis caused by *Salmonella* and its related functional mechanisms based on changes in intestinal microbiota and metabolomics.

To determine the relevant indicators of mouse serum samples, We first measured their pro-inflammatory and anti-inflammatory factors, including IL-1β and IL-6, which can stimulate cell proliferation and inhibit cell apoptosis, thus promoting inflammation [30]. IL-10 is a key immunosuppressive cytokine, and an increase indicates that the body’s immune function is limited [31]. The results show that *Salmonella* can increase pro-inflammatory cytokines such as IL-1β, IL-6, IL-8, and TNF-α, in the blood serum of mice, while anti-inflammatory cytokines IL-4 and IL-10 show a downward trend. TC can effectively reduce the incidence of diarrhea in mice by significantly lowering the concentrations of pro-inflammatory factors such as IL-1β, IL-6, IL-8, and TNF-α in serum and partially increasing the content of pro-inflammatory factors such as IL-4 and IL-10. It has good anti-diarrhea and anti-inflammatory effects. Microalgae and cyanobacteria [32], fucoidan [33], rosehip [34], and coptis [35] have all shown certain anti-inflammatory effects. Oxidative stress has been detected in the sera of mice, where it is considered an imbalance between the production of reactive oxygen species (ROS), eliminating protective mechanisms, which may lead to inflammation. Oxidative stress can activate various transcription factors, leading to the differential expression of genes involved in inflammation pathways. The inflammation caused by oxidative stress leads to many chronic diseases [36]. Malondialdehyde is the final product of free radical and lipid peroxidation reactions, reflecting the degree of lipid peroxidation in the body. SOD is an antioxidant enzyme that can catalyze free radicals into oxygen and hydrogen peroxide [37]. The above demonstrates that MDA and SOD contents in the body are often negatively correlated [38]. The results of this experiment are also consistent with this research trend. We thus conclude that compared with the model group, the TC group can significantly increase SOD levels and reduce MDA levels (*p* < 0.05), indicating that TC exerts a regulatory role through antioxidation. Furthermore, there is a certain positive correlation between the concentration of TC and its regulatory effect.

This study identified changes in the bacterial and fungal communities in the gut contents of mice infected with *Salmonella* and after TC treatment using 16S rRNA and ITS sequencing. The microbiome analysis indicated that *Salmonella* alters the structure and function of the gut microbiome, leading to ecological imbalance and infection. The changes in the main bacterial community imply that *Salmonella* increases the abundance of the *Candidatus_Saccharimonas*, *Enterorhabdus*, and *Alistipes* bacterial communities. Damage to the skin and mucous membranes of the body and gastrointestinal infections are often associated with the colonization of Candidatus in the skin or gastrointestinal tract [39]. Bruna Cristina dos Santos Cruz et al. [40] found that ProbiVSL#3 and yacon-based concentrates can reduce intestinal damage in a colitis-related model through a reduction in the abundance of the *Candidatus_Saccharimonas* bacterial community. There are many pathogenic bacteria in the *Enterorhabdus* genus, which may be the key pathogenic bacteria that induce enteritis. Wenjie Yi et al. [41] have also confirmed that lipid accumulation and inflammation in the liver in adult female offspring induced by maternal PFOS exposure in mice are closely related to changes in the abundance of *Enterorhabdus* genus bacteria. The *Alistipes* bacterial group plays a “double-edged sword” role in the intestinal microbiome, consisting of 13 species: *Alistipes finegoldii*, *Alistipes putredinis*, *Alistipes onderdonkii*, *Alistipes shahii*, *Alistipes indistinctus*, *Alistipes senegalensis*, *Alistipes timonensis*, *Alistipes obesi*, *Alistipes ihumii*, *Alistipes inops*, *Alistipes megaguti*, *Alistipes provencensis*, and *Alistipes massiliensis*. Bianca J Parker et al. [42] showed that *Alistipes* is pathogenic in colorectal cancer and associated with psychological symptoms of depression. Disruptions in the gut microbiome appear to play a role in determining the abundance of *Alistipes* in feces (e.g., non-alcoholic fatty liver disease, hepatic encephalopathy, and liver fibrosis). Our TC treatment significantly increased the *Alloprevotella* bacterial genus, which is a key player in the intestinal probiotic community. *Alloprevotella* can break down polysaccharides and produce short-chain fatty acids and other beneficial substances, providing energy and nutrients to the body. Additionally, *Alloprevotella* can maintain the integrity of the intestinal barrier. The intestinal barrier is an important line of defense in our bodies, preventing harmful substances from entering the bloodstream and protecting our health. *Alloprevotella* helps maintain the integrity and strength of this defense line by regulating the stability of the intestinal microbiome. Furthermore, it also plays a role in regulating the function of the immune system [43]. Baohai Liu et al. [44] found that Kuijieyuan Decoction can improve the intestinal barrier damage in ulcerative colitis by upregulating the abundance of the *Alloprevotella* bacterial community. Qian Xie et al. [45] also found that natural plant extracts from Huanglian and Magnoliae officinalis can repair the intestinal barrier of ulcerative colitis rats by increasing the abundance of beneficial bacteria such as *Alloprevotella*. Xiaofei Zhou et al. [46] found that coffee leaf tea extract can treat high uric acid kidney disease and its related negative effects on amino acid metabolism in rats by increasing the abundance of the *Alloprevotella* bacterial community. Changes in the abundance of the fungal genus Enterobacteriaceae in mice can be found by using ITS sequencing on their intestinal contents. The abundance of the fungal genus *Lodderomyces* was significantly upregulated in the model group compared with the control group (*p* < 0.05). A related study [47] showed that the fungal genus *Lodderomyces* is an emerging fungal pathogen that can cause fungal endocarditis [48], meningitis [49], vaginitis [50], and a series of inflammatory diseases; its invasive effect on epithelial and mucosal tissues may be the main reason for its pathogenicity [51]. The abundance of the *Trichosporon* fungal community significantly increased (*p* < 0.01) in the control group. These fungi are increasingly considered pathogens of superficial and invasive fungal diseases in biological bodies and belong to a class of opportunistic pathogens [52]. The *Trichosporon* fungus genus is prone to invading hosts with neutrophil deficiency [53], and it is more likely to cause invasive infections if the host is experiencing immunosuppression and decreased immunity [54]. The TC treatment group showed a significant increase in the *Saccharomyces* and *Penicillium* fungal populations (*p* < 0.01). The *Saccharomyces* fungal genus can participate in organic matter decomposition; it converts sugars, organic acids, and other organic substances into alcohol, carbon dioxide, and water, among other inorganic substances. These can then be utilized by other organisms, promoting the material cycling and energy flow within ecosystems. Meanwhile, the *Saccharomyces* fungal genus can also produce substances with antibacterial, antiviral, and other biological activities, which can then inhibit the growth and reproduction of harmful microorganisms and thus protect the host from pathogens [55]. The *Penicillium* genus can produce various antibacterial substances, such as penicillin and griseofulvin, with good anti-inflammatory and bactericidal activities [56]. Our analysis of changes in gut microbiome diversity was at the genus level. Future research directions will focus on finer levels and specific mechanisms. Thus, we conclude that TC extract can regulate the gut microbiome of mice, not only increasing its diversity but also regulating and improving the inflammatory response by upregulating the abundance of beneficial bacterial species such as *Alloprevotella* and *Saccharomyces* and fungi such as *Penicillium*.

By conducting a significant difference analysis of the metabolites between different groups, the following results were obtained: *Salmonella* significantly downregulated the prolactin signaling pathway, alanine, aspartate and glutamate metabolism, and D-amino acid metabolism (*p* < 0.01), causing changes in metabolites such as amino acids, steroid hormones, phospholipids, and carboxylic acids. The TC treatment group demonstrated an upregulating effect in most of the metabolic pathways, with significant upregulation of the Ras signaling pathway, long-term potentiation, MAPK signaling pathway, and Rap1 signaling pathway metabolic processes (*p* < 0.01). The Ras signaling pathway is related to cell proliferation, differentiation, apoptosis, and cancer occurrence. It also participates in immune system dysregulation, inflammation, and fibrosis in systemic sclerosis (SSc), as well as the destruction of synovial tissue and inflammatory diseases in rheumatoid arthritis (RA) [57]. The MAPK signaling pathway is widely involved in regulating various biological processes, such as cell growth, differentiation, death, and stress responses. It usually receives signals from cell surface receptors, such as receptor tyrosine kinases (RTKs) or G protein-coupled receptors (GPCRs), in response to signals from cytokines and environmental stressors. It is closely related to cell survival, inflammatory responses, and cell cycle regulation [58]. Numerous studies have shown that the Ras signaling pathway and the MAPK signaling pathway can serve as avenues for treating cancer and inflammatory diseases [59], and they are becoming popular targets for the development of targeted drugs [60,61]. In further research, we can explore the specific mechanisms by which TC extracts regulate gut microbial dysbiosis caused by *Salmonella* in mice by validating these metabolic pathways, and we can also move toward targeted drug research.

## 5. Conclusions

This study successfully established a mouse model of intestinal salmonellosis caused by enteric *Salmonella*, in which bacteria invade the intestinal tissue, causing tissue damage and triggering an inflammatory response and oxidative stress, which alter the body’s internal environment equilibrium. This leads to metabolic disorders in the intestine and changes in the diversity and abundance of various bacterial and fungal species in the intestinal microbiome. Consequently, the TC extract alleviated the intestinal inflammation caused by *Salmonella* by inhibiting the release of pro-inflammatory factors, upregulating the level of anti-inflammatory factors, and enhancing the body’s antioxidant capacity to improve oxidative stress and lipid peroxidation-induced damage and index changes. The TC extract can also regulate the relative abundance of intestinal microbial bacteria and fungi, change the metabolites on the intestinal epithelial cell membrane, repair the damaged intestinal barrier, and balance the release of harmful metabolites through adjusting or downregulating related metabolic pathways, indirectly affecting the structure of intestinal microbial community, thus promoting the survival of probiotic bacteria. The TC extract has good therapeutic effects against *Salmonella* infection and can effectively treat bovine bacterial diarrhea. In our next study, the specific mechanisms of action of the bacterial strains and metabolites will be elucidated, in order to explore and verify the specific pathways and target proteins of TC extract in treating *Salmonella* infection. This will provide data support for the research and development of natural plant drugs and pharmacological studies.

## Data Availability

The original contributions presented in this study are included in the article, and further inquiries can be directed to the corresponding author.
